# Hospital-wide survey of clinical experience with artificial intelligence applied to daily chest radiographs

**DOI:** 10.1371/journal.pone.0282123

**Published:** 2023-03-02

**Authors:** Hyun Joo Shin, Seungsoo Lee, Sungwon Kim, Nak-Hoon Son, Eun-Kyung Kim

**Affiliations:** 1 Department of Radiology, Research Institute of Radiological Science and Center for Clinical Imaging Data Science, Yongin Severance Hospital, Yonsei University College of Medicine, Yongin, Gyeonggi-do, Republic of Korea; 2 Center for Digital Health, Yongin Severance Hospital, Yonsei University College of Medicine, Yongin, Gyeonggi-do, Republic of Korea; 3 Department of Radiology, Research Institute of Radiological Science and Center for Clinical Imaging Data Science, Severance Hospital, Yonsei University College of Medicine, Seoul, Republic of Korea; 4 Department of Statistics, Keimyung University, Daegu, Republic of Korea; University of Maryland College Park, UNITED STATES

## Abstract

**Purpose:**

To assess experience with and perceptions of clinical application of artificial intelligence (AI) to chest radiographs among doctors in a single hospital.

**Materials and methods:**

A hospital-wide online survey of the use of commercially available AI-based lesion detection software for chest radiographs was conducted with all clinicians and radiologists at our hospital in this prospective study. In our hospital, version 2 of the abovementioned software was utilized from March 2020 to February 2021 and could detect three types of lesions. Version 3 was utilized for chest radiographs by detecting nine types of lesions from March 2021. The participants of this survey answered questions on their own experience using AI-based software in daily practice. The questionnaires were composed of single choice, multiple choices, and scale bar questions. Answers were analyzed according to the clinicians and radiologists using paired t-test and the Wilcoxon rank-sum test.

**Results:**

One hundred twenty-three doctors answered the survey, and 74% completed all questions. The proportion of individuals who utilized AI was higher among radiologists than clinicians (82.5% vs. 45.9%, p = 0.008). AI was perceived as being the most useful in the emergency room, and pneumothorax was considered the most valuable finding. Approximately 21% of clinicians and 16% of radiologists changed their own reading results after referring to AI, and trust levels for AI were 64.9% and 66.5%, respectively. Participants thought AI helped reduce reading times and reading requests. They answered that AI helped increase diagnostic accuracy and were more positive about AI after actual usage.

**Conclusion:**

Actual adaptation of AI for daily chest radiographs received overall positive feedback from clinicians and radiologists in this hospital-wide survey. Participating doctors preferred to use AI and regarded it more favorably after actual working with the AI-based software in daily clinical practice.

## Introduction

Artificial intelligence (AI) has rapidly found use in medical imaging [1, 2]. Through the development and validation of AI-based algorithms, new commercially available software systems have emerged, such as those for chest radiographs, mammography, or bone age radiographs [3–7]. Apart from validating the performance of each AI-based solution, recent efforts have drawn attention for proving the clinical efficacy of AI in actual medical processes [1, 8, 9].

Several studies have reported positive feedback from radiologists and residents concerning the adaptation of AI in clinical practice [10–14]. They found that most radiologists agreed that more research and application of AI are necessary. Other surveys have also highlighted positive attitudes towards AI use among clinicians [15, 16]. However, unlike the grand expectations made with the first introduction of AI, the number of radiologists truly using AI for daily imaging interpretations is small [13]. Therefore, it is important to understand what doctors really experience when incorporating AI-based methods to interpret radiographs in order to determine the future direction of AI use in medicine.

Recently, AI-based lesion detection algorithms were introduced for chest radiographs and approved due to acceptable diagnostic performance shown for various diseases, such as pneumonia, tuberculosis, and lung nodules [7, 17–21]. However, approval of clinical application of commercially available software remains limited [2, 8]. A recent consensus among thoracic radiologists on the utilization of AI-based medical devices found that AI could assist the interpretation work of radiologists and support the decision-making process of clinicians when radiologists are not available [4]. They concluded that the effective and convenient placement of AI-based devices in clinical environments is essential to maximize the merits of AI in medicine [4]. As our hospital has utilized an AI-based device for all chest radiographs since March 2020 [20, 22], we wanted to know what doctors thought about the actual integration of AI in daily practice. Therefore, we performed a hospital-wide survey to document how the AI-based device has impacted the clinical process and what impressions clinicians and radiologists formed after utilizing it in real situations.

The purpose of this study was to understand the effect of clinically applying AI regularly to daily chest radiographs through a hospital-wide survey of clinicians and radiologists.

## Materials and methods

### Subjects

The Institutional Review Board (IRB) of Yongin Severance Hospital approved this prospective study (IRB number 9-2021-0073). All doctors in our hospital received an e-mail containing a link for the online survey in July 2021, and the study participants included those who freely decided to take the survey anonymously within 2 weeks after receiving the e-mail. Sample size calculation or sampling was not performed in this survey study, and we included doctors who were willing to participate in this survey autonomously. Among the participants, written informed consent was obtained before they began to answer the 25 questions of the online survey autonomously under the guidance of the IRB of our hospital. The online surveys were uploaded in a web-based format (SurveyMonkey.com). All study methods were in accordance to the Consensus-based Checklist for Reporting of Survey Studies guidelines. All participants were either clinicians or radiologists who worked in our hospital during 2020 or 2021. The complete question forms are provided in S1 File. Participants answered questions concerning their clinical experience with AI-based lesion detection software for chest radiographs. Questions were designed to gather information on basic demographics, experience with AI, actual individual utilization status of AI, and preferences and attitudes toward AI-based software after actual usage. The questionnaires were composed of a single choice, multiple choices, and scale bar questions, and there were no open-ended questions. The composition of questionnaires and way of presenting answers were constructed and validated under the guidance of a statistical expert to analysis results in an objective way as much as possible and to overcome the radical subjectivity of a survey study.

### Use of AI-based lesion detection software for chest radiographs

**Fig 1:**
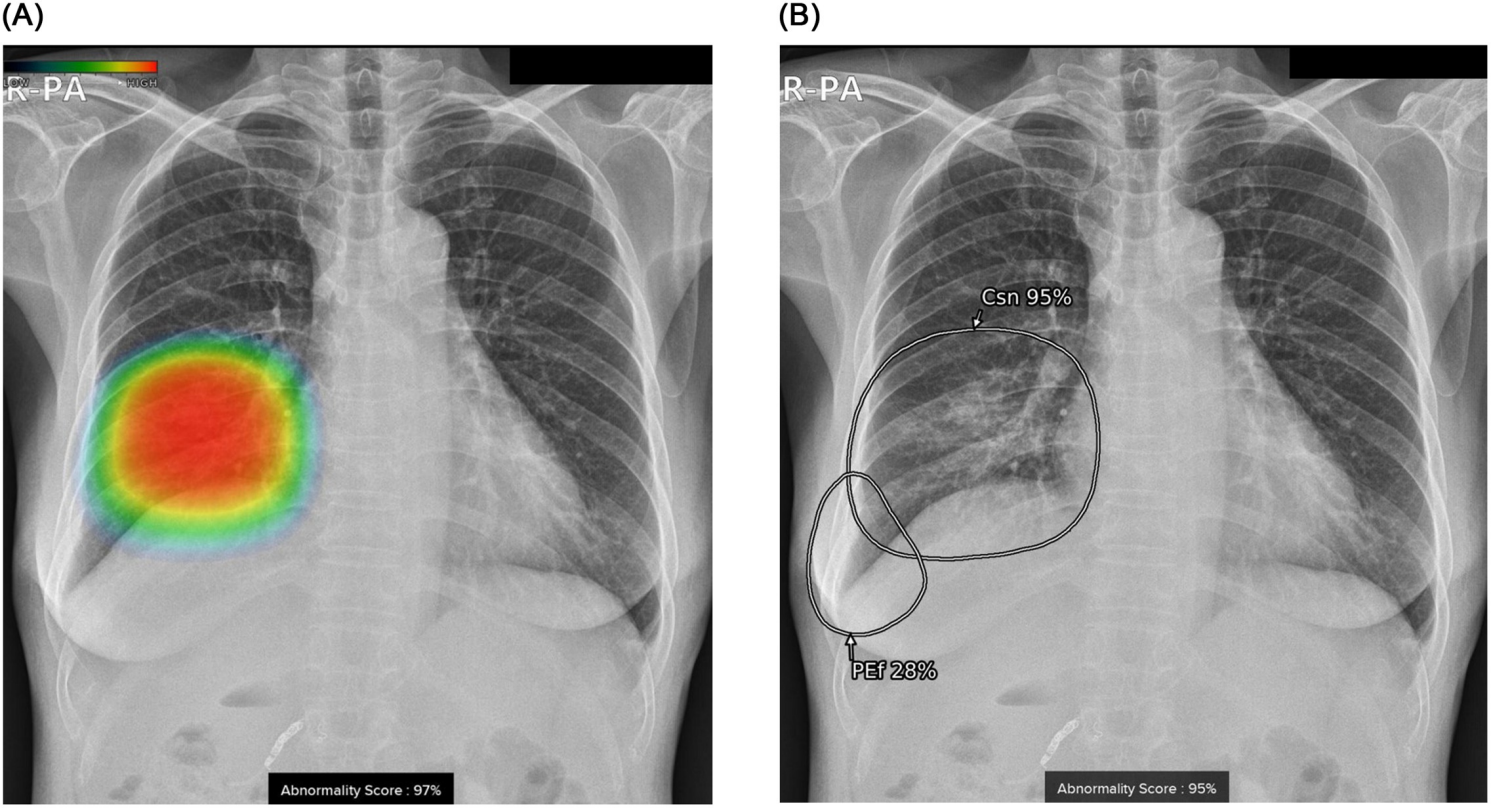
Chest radiographs of a 57-year-old female with pneumonia and right pleural effusion. Images are results analyzed with (A) version 2 and (B) version 3 of the AI-based lesion detection software. (A) Version 2 can detect and display three types of lesions (consolidation, nodule, and pneumothorax) with a color heatmap and total abnormality score. (B) Version 3 can detect and display nine types of lesions (six additional types of the lesion in addition to the three lesions detected in version 2) with a grayscale heatmap and abnormality score for each lesion. Note the right pleural effusion that was additionally detected and displayed with version 3 of the software.

In our general hospital, commercially available AI-based lesion detection software (Lunit INSIGHT CXR, versions 2 and 3, Lunit Inc., Korea.) has been run on all chest radiographs from patients over 18 years old since March 2020. This ResNet34-based software was developed and approved for adult chest radiographs with the anteroposterior and posteroanterior view [23]. Detailed information about the integration process of AI for chest radiographs was well introduced in a recent study [20]. In our hospital, version 2 of the abovementioned software was utilized from March 2020 to February 2021 and could detect three types of lesions (nodule, consolidation, and pneumothorax) (Fig 1A). From March 2021, version 3 was utilized for chest radiographs by detecting nine types of lesions (nodule, consolidation, pneumothorax, pneumoperitoneum, fibrosis, atelectasis, cardiomegaly, calcification, and pleural effusion). This was an upgraded version including three types of the lesions detected in version 2, and these two versions were not from different software. Under the guidance of the radiology department of our hospital, version 2 was used from March 2020 to February 2021, and version 3 replaced version 2 from March 2021 and was used for all chest radiographs since then. Therefore, the users of version 2 and 3 were not different. The abbreviations and abnormality scores of each lesion are displayed with an additional grayscale heatmap at the lesion location in version 3 (Fig 1B).

**Fig 2:**
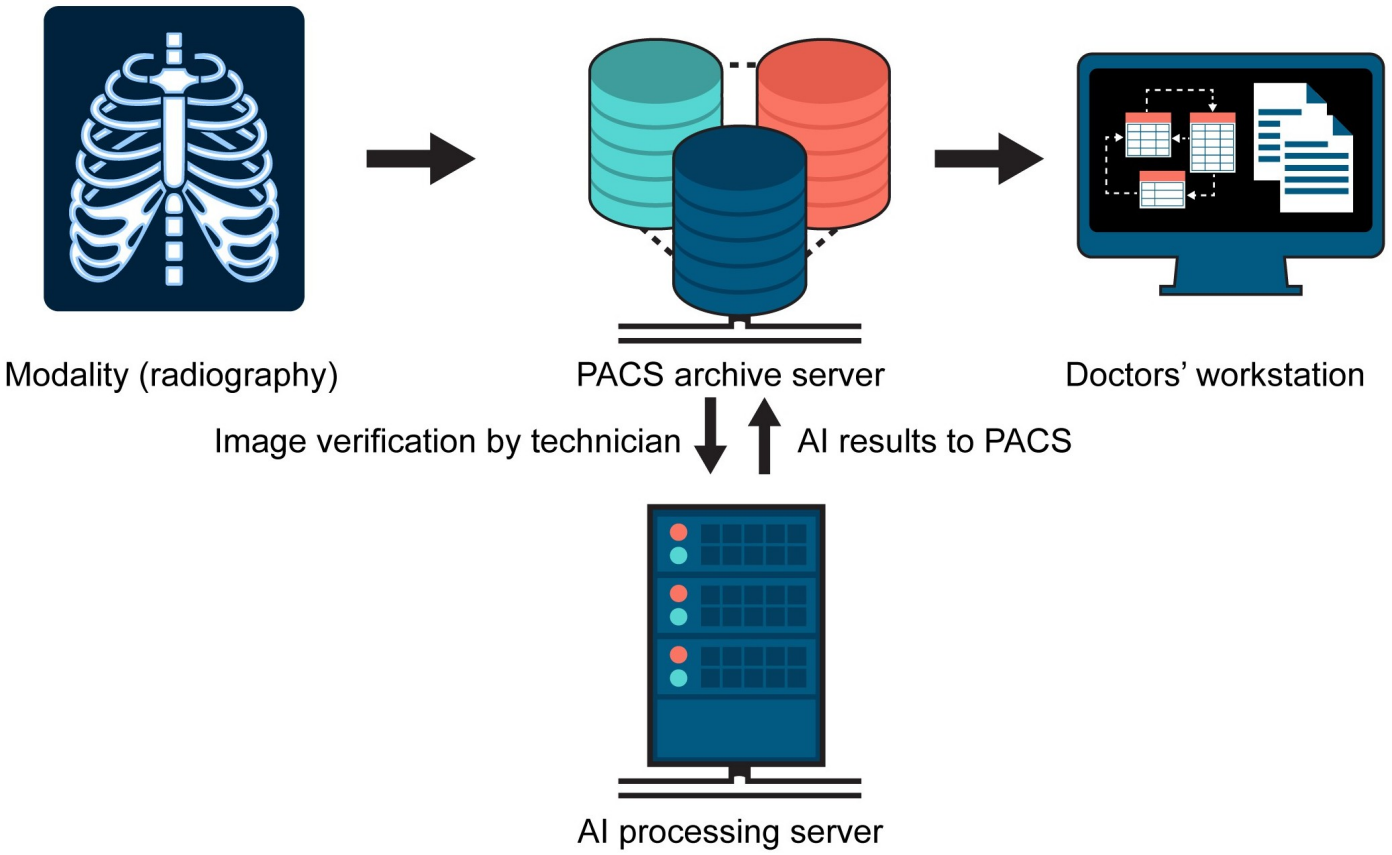
Workflow for utilizing the AI results in the PACS system.

The workflow for utilizing the AI system in our picture archiving and communication system (PACS) is shown in Fig 2. As soon as the chest radiographs were verified by the radiographers, the images were automatically sent to the AI processing server. PACS automatically retrieved the AI results and made them accessible on the radiologists’ or clinicians’ workstations. PACS viewer software (Zetta PACS, Taeyoung soft Co. Ltd., Korea) presented the total abnormality score on a worklist with several display options for the results. Contour maps were attached as separate captured images following the original radiographs. An abnormality score of 0.15 was the cutoff for visualization on the contour maps for each lesion according to the vendors’ guidelines and other studies [23–26]. Through this process, doctors could refer to the analyzed images simply by scrolling down from the original radiographs whenever they wished to refer to the AI results. Therefore, the participants of this survey answered questions on their own experience using AI-based software in daily practice from March 2020.

### Statistical analysis

SAS software version 9.4 (SAS Institute Inc., Cary, NC, USA) was used for the statistical analysis. Data are presented as means with standard deviations and medians with interquartile ranges (Q1, Q3). The two-sample t-test or Fisher’s exact test was used for group comparison. The paired t-test was used for comparisons within groups, and the Wilcoxon rank-sum test was used for comparison between groups. P-values less than 0.05 were considered as statistically significant.

## Results

### Demographics

**Fig 3:**
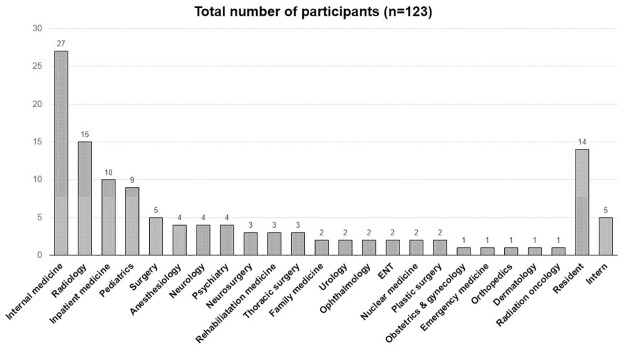
Subspecialties of the participants.

Among 194 doctors in our hospital, a total of 123 doctors (63.4%) answered the survey, and 91 (clinicians: radiologists = 78:13) completed it in full. The mean proportion of completed questions among all given questions was 79%, and the survey took an average 7 minutes, 48 seconds to complete. Basic participant demographics are summarized in Table 1. Most doctors of our hospital were board-certified staff because our general hospital newly opened in March 2020. Except for several interns and residents in family medicine, there were no trainees in most departments, including radiology, during the study period. Thus, board-certified doctors accounted for 83.7% of the survey participants, and the remaining 16.3% were residents or interns. The subspecialties of the participants are displayed in Fig 3. Among the radiologists, there was only one thoracic radiologist. However, in our hospital, all chest radiographs are interpreted by board-certified radiologists regardless of their subspecialty, because the number of chest radiographs that need to be interpreted are high. Radiologists are expected to read a minimum of 500 radiographs every month. The mean number of adult chest radiographs obtained per month from March 2020 to July 2021 was 6,849. The proportion of doctors who had previous experience with AI-based education was significantly smaller among clinicians than radiologists (27.6% vs. 93.3%, p<0.001). The proportion of doctors who had experience with AI-based research was also smaller among clinicians (21% vs. 46.7%, p = 0.049). When asked about overall personal experience with AI (Table 2), clinicians and radiologists showed significant increases in experience after March 2020, compared to before (all, p<0.005), and this increase was more pronounced among radiologists (38% vs. 54.6%, p = 0.01).

**Table 1:** Participant demographics.

Survey Questions		Clinicians (n = 108)		Radiologists (n = 15)		p-value
		n	%	n	%	
Sex	Male	69	63.9	8	53.3	0.429^b^
	Female	39	36.1	7	46.7	
Age	20–29	10	9.3	0	0.0	0.612^c^
	30–39	55	50.9	8	53.3	
	40–49	30	27.8	4	26.7	
	Greater than their 50’s	13	12.0	3	20.0	
Title	Assistant professor	52	48.2	9	60.0	0.281^c^
	Associate professor	21	19.4	3	20.0	
	Professor	15	13.9	3	20.0	
	Resident, intern	20	18.5	0	0.0	
Location of dedicated patients ^a^	ER	56	51.9	.	.	.
	ICU	36	33.3			
	Inpatient	78	72.2			
	Outpatient	71	65.7			
Experience with AI-based education	Yes	29	27.6	14	93.3	<0.001^c^
	No	76	72.4	1	6.7	
Experience with AI-based research	Yes	22	21.0	7	46.7	0.049^c^
	No	83	79.1	8	53.3	

^a^Multiple choice,

^b^Two-sample t-test,

^c^Fisher’s Exact test.

Abbreviations: AI = artificial intelligence, ER = emergency room, ICU = intensive care unit.

**Table 2:** Scale bar questions concerning the utilization of chest radiographs.

Survey Questions		Clinicians (n = 78)					Radiologists (n = 13)					p-value^b^
		Mean	SD	Median	Q1	Q3	Mean	SD	Median	Q1	Q3	
Proportion of chest radiographs among all utilized images in a day		45.2	29.3	50	15	70	18.8	18.8	12	7	30	0.003
Proportion of cases referring to AI results among all utilized chest radiographs in a day		45.9	41.1	30	7	100	82.5	29.9	100	85	100	0.008
Overall experience with AI	~2020.2	13.5	18.8	1	0	21	21.3	28.2	1	0	48	0.541
	2020.3~	38.0	25.5	40	20	51	54.6	23.0	51	31	75	0.010
	Difference	24.4	23.0	21	5	43	33.3	27.4	31	23	51	0.066
	p-value^a^	<0.001					<0.001					

^a^Paired t-test (within Group),

^b^Wilcoxon rank-sum test (between groups).

Data are presented as means with standard deviations (SDs) or medians with interquartile ranges (Q1-Q3).

Abbreviations: AI = artificial intelligence.

### Utilization of AI for chest radiographs

**Fig 4:**
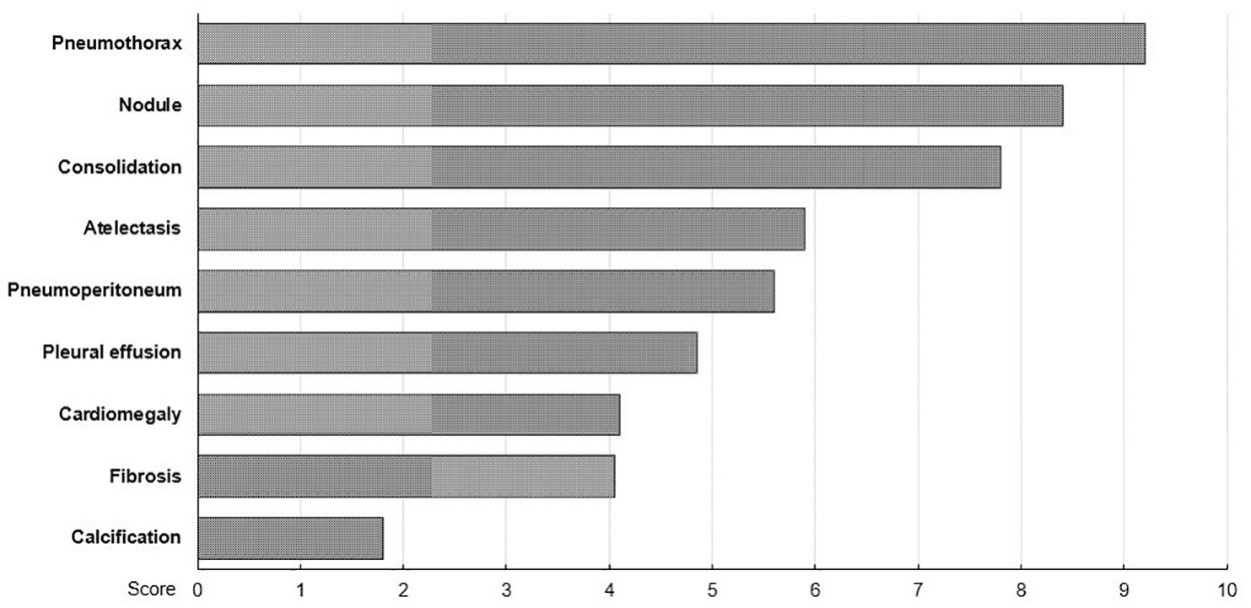
Ranking the most useful finding among the nine types of lesions analyzed by AI.

As shown in Table 2, the proportions of chest radiographs among all utilized imaging studies in daily practice were 45.2% for clinicians and 18.8% for radiologists (p = 0.003). However, the proportion of chest radiographs for which doctors utilized AI results in a day was significantly higher among radiologists than clinicians (45.9% vs. 82.5%, p = 0.008). When participants were asked to pick the location where they thought the AI results were put to best use, they answered the emergency room (ER), outpatient unit, inpatient unit, and intensive care unit (ICU) in descending order. When radiologists were asked to choose the most useful finding among the nine types of lesions assessed by AI, they answered pneumothorax, nodules, consolidation, atelectasis, pneumoperitoneum, pleural effusion, cardiomegaly, fibrosis, and calcification in descending order (Fig 4). When asked to choose between versions 2 and 3, 83.3% of clinicians and 84.6% of radiologists preferred version 3 of the AI-based software. However, on the method of display, clinicians (59%) preferred the color heatmap, while radiologists (69%) preferred the grayscale heatmap.

### Multiple-choice questions assessing AI experience

Participants were allowed to choose multiple answers for each question (Table 3). When asked why they referred to AI results, 74.7% of the participants answered that they referred to AI results with the belief that it would help lower the risk of missed diagnoses. About 35.2% of the participants answered it was because it was easy to refer to the AI results on PACS, 27.5% said it had become routine to check the AI results, and 19.8% said they referred to the AI results for their relative accuracy.

**Table 3:** Multiple-choice questions concerning AI experience.

Survey Questions		Clinicians (n = 78)	Radiologists (n = 13)	Overall, %
		n (%)	n (%)	
Reasons for referring to the analysis results of the AI program	Relatively accurate AI results	13 (16.7)	5 (38.5)	19.8
	Lowers the risk of missing lesions	55 (70.5)	13 (100)	74.7
	User-friendly method that makes checking AI results easy	26 (33.3)	6 (46.2)	35.2
	Has become routine to check the AI results	19 (24.4)	6 (46.2)	27.5
AI results that were mainly referred to	Total abnormality score	36 (46.2)	9 (69.2)	49.5
	Lesion type (abbreviation)	38 (48.7)	4 (30.8)	46.2
	Lesion location (ROI)	52 (66.7)	11 (84.6)	69.2
	Abnormality score (per lesion)	20 (25.6)	3 (23.1)	25.3
Best advantage of the AI-based software	Shortens decision times	35 (44.9)	8 (61.5)	47.3
	Enables lesion detection	49 (62.8)	11 (84.6)	65.9
	Discrimination of normal and abnormal lesions	45 (57.7)	9 (69.2)	59.3
	Differential diagnosis of lesions	12 (15.4)	0 (0)	13.2
	Triage of radiographs for reading sessions	4 (5.1)	2 (15.4)	6.6
AI results that would be welcomed if available after future developments in technology	Addition of readable lesion types	15 (19.2)	1 (7.7)	17.6
	Increased diagnostic accuracy for lesion detection	40 (51.3)	9 (69.2)	53.8
	Comparison function for lesions	46 (59)	8 (61.5)	59.3
	Alarm system for urgent conditions	44 (56.4)	5 (38.5)	53.8
	Expansion of the applicable age range	11 (14.1)	6 (46.2)	18.7
	Broader application to imaging other than chest imaging	25 (32.1)	3 (23.1)	30.8

Abbreviations: AI = artificial intelligence, ROI = region-of-interest.

When surveying to which results the participants mainly referred, 69.2% of the participants answered the location of the lesion that was displayed on chest radiographs, 49.5% selected the total abnormality score of each radiograph, 46.2% chose per lesion abbreviations, and 25.3% selected per lesion abnormality score. When asked for what each regarded as the most useful advantage of incorporating AI, 65.9% selected lesion detection, 59.3% chose discrimination of normal and abnormal radiographs, 47.3% chose quicker decision making, 13.2% selected differential diagnosis of lesions, and 6.6% selected triage of reading.

When asked for which function or information they wanted to be developed for AI in the future, 59.3% of the participants chose a comparison function that would automatically compare images to previous radiographs, such as the comparison of pleural effusion or pneumothorax. About 53.8% selected increased diagnostic accuracy for lesion detection and also an alarm system for urgent lesions on radiographs. About 30.8% chose expanded use to different types of radiographs other than chest imaging, 18.7% selected broadening the applicable age to patients less than 18 years old, and 17.6% selected increasing the types of lesions detectable on chest radiographs.

### Scale-bar questions assessing AI experience

We asked to subjectively choose percentages from 0 to 100% or -50 to +50% using the sliding bar function on the website to compare perceptions of AI before and after use thereof (Table 4). As many as 21% of clinicians and 16% of radiologists said that they had changed their own reading results after referring to the AI results (p = 0.727). Clinicians and radiologists said their trust levels for AI were about 64.9% and 66.5%, respectively (p = 0.759).

**Table 4:** Scale-bar questions of AI experience.

Survey Questions	Scales (%)	Clinicians (n = 78)						Radiologists (n = 13)						p-value^a^
		n	Mean	SD	Median	Q1	Q3	n	Mean	SD	Median	Q1	Q3	
Extent of changing one’s own reading results after referring to the AI results	0~100	78	20.9	23.0	17	1	30	13	15.9	14.5	12	5	25	0.727
Subjective trust levels for the AI results	0–100	78	64.9	20.9	70	50	80	13	66.5	20.8	70	61	81	0.759
Influence on the reading time of chest radiographs	-50~+50	78	-10.7	25.0	-16	-25	0	13	-14.5	12.1	-18	-25	-10	0.869
Influence on the number of reading requests	-50~+50	78	-12.2	17.2	0	-22	0	13	-23.5	17.3	-22	-36	-16	0.026
Diagnostic accuracy of their reading	-50~+50	78	23.8	14.8	21	13	30	13	28.0	10.0	29	25	30	0.150
Perceptions on AI-based medical devices	-50~+50	78	27.6	17.0	28	15	45	13	27.7	11.2	26	21	30	0.946
Perceptions on the future use of AI	-50~+50	78	32.1	15.5	30	20	50	13	37.5	15.4	45	30	50	0.289

^a^Wilcoxon rank-sum test (between groups)

Data are presented as means with standard deviations (SDs) or medians with interquartile ranges (Q1-Q3).

Abbreviations: AI = artificial intelligence.

We used the -50 to +50% scales when the answers could be binary, such as a negative or positive attitude, or decreased or increased results according to the participants’ perceptions about AI usage. When asked how the AI results would affect the reading times of chest radiographs, clinicians and radiologists said that they thought AI helped to reduce reading times and the number of reading requests for chest radiographs. In comparison of answers between clinicians and radiologists, the only significant difference was observed in the question concerning reading requests. Radiologists thought that reading requests for chest radiographs were reduced more than indicated by clinicians (-23.5% vs. -12.2%, p = 0.026). In addition, participants thought that diagnostic accuracy would increase after using AI, and they were more positive about AI-based medical devices after using them in clinical practice. Both clinicians and radiologists felt positive about the future usage of AI.

### Comparison of staff and trainees among clinicians

The overall answers of trainees among clinicians are summarized in S2 File. There were no significant differences in perceptions of AI-based devices between staff and trainees among clinicians.

## Discussion

Our study showed that about 46% of clinicians and 83% of radiologists referred to the analyzed results of the AI-based software in daily clinical practice for chest radiographs. The most common reason for using the software was to reduce missed diagnoses, and the second reason was because the software made it easy to utilize the AI results on PACS. Interestingly, 28% of participants answered that referring to the AI results had become routine during their readings of chest radiographs. This shows that presenting analyzed results with an efficient and user-friendly interface is critical for the successful adaptation of AI into the clinical process. It is notable that many doctors now routinely refer to AI in their everyday workflow as this gives us a glimpse of what full adaptation of AI can mean for radiology in the future.

The most useful location for utilizing the AI results was the ER and the outpatient unit. This indicates that the AI-based software was more useful in locations that require urgent decisions to be made that cannot wait a radiologist’s reading. Among the various lesion types, urgent or important lesions were thought to reap the benefits of AI results, with pneumothorax, nodule, and consolidation in decreasing order, even though the pneumoperitoneum was ranked lower. This result may be influenced by the incidence of detected lesion types to depict the most useful finding by the participants. Our results suggest that AI-based software can be adapted to chest radiographs effectively. This is important because chest radiographs are still one of the first imaging tools used to guide future treatment and that AI can help depict urgent conditions on chest radiographs even in situations where it is difficult to get a reading right away from a radiologist who is already dealing with a large number of daily images [27]. This means AI can help clinicians and radiologists to catch important diseases and can be adapted for critical diseases first and more effectively [27].

Concerning their experience with AI and the buildup of trust in the results from AI, clinicians and radiologists said they had changed their own reading results after referring to AI, with these changes thought to occur in 21% and 16% of cases, respectively. Doctors rated the trust levels for AI results as 64.9% and 66.5% for clinicians and radiologists, respectively. The AI-based software had a tendency to reduce reading times and the number of reading requests for chest radiographs. Concerning the perception for AI between radiologists and clinicians, radiologists thought reading requests for chest radiographs were reduced more than clinicians. This would be because compared to the various number of clinicians who give reading requests, a limited number of radiologists had to read chest radiographs for the entire hospital. This could make radiologists feel the effect of AI more on reducing reading requests after integrating AI. In addition, participants answered that using the software increased diagnostic accuracy and that they regarded AI in a more positive light after actual usage. They were optimistic about the future usage of AI. Another interesting thing to note is that doctors seem to have truly accepted the adaptation of AI-based software for daily chest radiographs based on the overall positive feedback collected through the survey responses. This study was meaningful because it gives a broad picture of the actual clinical effect of AI and how it is perceived through the eyes of a relatively large number of doctors from an entire hospital.

In a recent survey of trainees and fellows including radiologists from two nations, 60% of the participants answered that AI would impact clinical reality in less than 5 years, especially for screening disease and reducing the time needed for monotonous work [15]. However, 80.9% of clinicians answered in the same survey that they did not have any actual experience with AI despite these high expectations [15]. Still, AI-based methods have been developed and validated for various diseases, but mostly in a retrospective manner, and there are not many conclusions based on external validation or genuine clinical use [3, 8, 19, 28–30]. In another large survey in Europe, the degree of AI-based knowledge inversely affected fear about AI and affected perceptions about AI [10]. Another survey by the same research group showed that less knowledge and ethical issues could interrupt the wide adaptation of AI in clinical practice [12]. Despite implementation of AI being in its beginning stages, most studies showed a positive attitude toward AI in radiology and acceptance of its inevitable adaptation to medical imaging [11, 15, 23]. In a recent consensus statement, expert chest radiologists concluded that AI-based medical devices could help clinicians make decisions when radiologists are not promptly available and that AI could act as an assistant for radiologists [4]. Creating an effective clinical environment is a key factor for the successful adaptation of AI-based devices in medicine [4, 20, 22, 31]. Our study is meaningful because it demonstrates how commercially available AI-based software has actually been implemented in clinical practice and integrated successfully from an entire hospital-wide perspective. Chest radiographs are commonly performed and utilized for all subspecialties. Therefore, integration of AI on chest radiographs could broadly impact clinical practices [24, 26, 32–34], and our survey demonstrated how doctors are affected after AI is integrated into the daily imaging process in March 2020. In addition, our study showed that the clinicians and radiologists of our institution regarded AI more favorably after working with the AI-based software.

Our study has several limitations. First, according to our hospital’s characteristics, we could not compare actual experience before and after the adaptation of AI because our hospital adopted this software from its first opening. In addition, we could not include radiology trainees because most departments of our hospital did not have them at that time and doctors of relatively young age consisted a large proportion of the whole medical team (about 59.4% of participants were in their twenties or thirties), compared to other hospitals. The responses to the survey questions were based on each doctor’s personal experience before and after working at our hospital. To obtain qualified and objective results, we asked the participants of the survey to answer using mostly 0–100% or -50-+50% scales for dedicated answers. Second, the actual effect of AI on workflow and outcomes was not assessed quantitatively in this survey study. According to the nature of survey study, this was mainly based on participants’ perceptions of how accurate the AI was. However, because the adapted period was not short and there are very few surveys conducted by various doctors of different departments in an entire hospital unit, we thought that the influence of AI on clinical practice would be reflected in this survey and that this study would be of interest to the readers in the recent status of AI for radiology. Because this survey was performed in 2021, the doctors’ acceptance of and experience with AI could have changed as time passes. Further continuous studies demonstrating how AI has changed actual workflows and the perception of doctors and has influenced clinical outcomes are needed as experience accumulates. We are in the process of demonstrating the effect of AI on diagnostic accuracy or reading time to justify these survey results in an objective way and hope to confirm our findings with quantitative results in the next step of our research.

## Conclusions

The real adaptation of an AI-based software for daily chest radiographs received overall positive feedback from clinicians and radiologists in this hospital-wide survey. They preferred to use AI to reduce missed diagnoses, and the most useful location for utilizing the AI was the ER. The survey participants thought that AI could help them to catch important diseases and be adapted for critical diseases effectively. The clinicians and radiologists regarded AI more favorably after actual working with the AI-based software in daily clinical practice.

## Supporting information

S1 File Survey questions for clinicians concerning their experience with AI-based lesion detection software for chest radiographs. (DOCX)

S2 File Comparison of responses between staff and trainees among clinicians. (DOCX)

S1 Data Data file. (XLSX)

## Abbreviations

AI: artificial intelligence
ER: emergency room
ICU: intensive care unit
PACS: picture archiving and communication system
SD: standard deviation
