# Supplementary material for: Hospital-wide survey of clinical experience with artificial intelligence applied to daily chest radiographs
Source: PLoS One. 2023 Mar 2;18(3):e0282123. doi: 10.1371/journal.pone.0282123 (PMC9980810; doi:10.1371/journal.pone.0282123)
Supplement: S1 File — (DOCX) [file pone.0282123.s001.docx]

**S1 appendix file.**

Survey questions **for clinicians** about their experience with the AI-based lesion detection software for chest radiographs

Do you agree to participate in the following survey study?

a. Yes

b. No

If you agree, please respond to the following questions.

[Basic Demographics]

1. What is your sex?

a. Male

b. Female

2. Which of the following age groups do you belong to?

a. 50-59

b. 40-49

c. 30-39

d. 20-29

3. Please write the department in which you specialize in.

( )

4. What position do you hold in your department?

a. Professor

b. Associate professor

c. Assistant professor

e. Fellow

f. Resident

g. Intern

5. When did you start working at our institution?

a. In 2020

b. In 2021

6. Where are the patients you are generally dedicated to located? (Please check all that apply.)

a. ER

b. ICU

c. Inpatient Unit

d. Outpatient Unit

[Experience with AI]

7. Do you have any educational experience with artificial intelligence?

a. Yes

b. No

8. Do you have any research experience with artificial intelligence?

a. Yes

b. No

9. How much experience did you have with artificial intelligence-based medical devices before working at our hospital? (Please select a percentage compared to your current experience load by moving the round button. For example, if you had no experience, choose 0%. If you work with the devices at a similar load, choose 100%.)


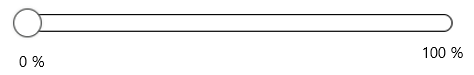


10. How much experience did you gain with artificial intelligence-based medical devices after working at our hospital? (Please select a percentage by moving the round button. For example, if you did not work with artificial intelligence-based medical devices during the study period at all, choose 0%.)


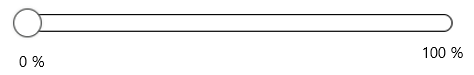


[Utility of the AI-based software]

11. What proportion of all imaging tests that you are responsible for during a single workday are chest radiographs? (Please select the appropriate percentage by moving the round button.)


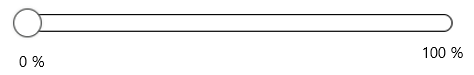


12. Of the chest radiographs you review each day, what percentage are interpreted using the AI-based software? (Please select the appropriate percentage by moving the round button.)


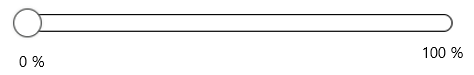


13. Why do you refer to the analysis results of the AI program?

a. Because the AI results are relatively accurate.

b. Because their information can be used to lower the risk of missing lesions.

c. Because the user-friendly interface makes it easy to check the AI results.

d. Because it has become routine to check the AI results.

14. If you refer to the analysis results of the AI program, please indicate all the results you choose. (Multiple choice)

a. Total abnormality score (displayed per X-ray)

b. Type of lesion detected (abbreviation)

c. Lesion location (ROI)

d. Abnormality score of each detected lesion

15. How much trust do you have for the analysis results of the AI program? (Please choose the appropriate percentage by moving the round button.)


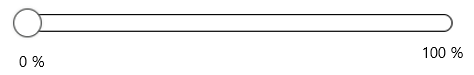


16. Please indicate to what extent your own chest X-ray readings have changed after referring to the AI results. (Please select the appropriate percentage by moving the round button.)


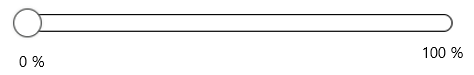


17. How are the chest X-ray reading times affected by this AI program?

(-50%: decreased, +50%: increased)


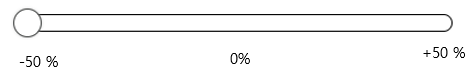


18. What do you consider the best advantage of the AI program?

a. Shortens decision times.

b. Helps detect lesions.

c. Helps discriminate normal and abnormal lesions.

d. Helps the a differential diagnosis of lesions.

e. Enables triage of radiographs for reading.

19. Did the number of reading requests for chest radiographs change after the AI program was applied in our hospital? (-50%: decreased, +50%: increased)


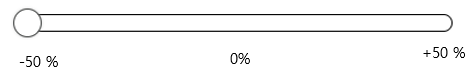


20. Please mark the circle to indicate how much the AI program has helped clinicians read the chest radiograph readings of patients in the following locations.


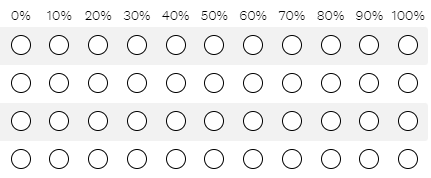


Outpatient

Inpatient

ER

ICU

21. Which version of the AI program do you prefer, version 2 or 3?

a. Version 2

b. Version 3

22. Which image do you prefer, the color heatmap or grayscale heatmap?

a. Color heatmap

b. Grayscale heatmap

23. What other information would you like to see in the AI results in the future? (Multiple choice)

a. Addition of readable lesion types

b. Increased accuracy for lesion detection

c. Comparison function that analyzes degrees of change compared to previous images

d. Alarm system for urgent conditions

e. Expansion of the applicable age group

f. Broader application to imaging other than chest imaging

24. After using the AI analysis program, how have your perceptions on AI-based medical devices changed? (-50%: negative, +50%: positive)


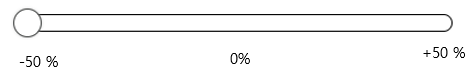


25. Do you plan to use AI diagnostic programs for reading in the future? (-50%: negative, +50%: positive attitude)


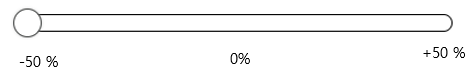


Thank you for participating in our survey.

**Supplementary file 1-2.**

Survey questions **for radiologists** about their experience with AI-based lesion detection software for chest radiographs

Do you agree to participate in the following survey study?

a. Yes

b. No

If you agree, please respond to the following questions.

[Basic demographics]

1. What is your sex?

a. Male

b. Female

2. Which of the following age groups do you belong to?

a. 50-59

b. 40-49

c. 30-39

d. 20-29

3. Please select the field of radiology that you specialize in.

a. gastrointestinal, genitourinary radiology

b. thoracic, cardiac radiology

c. neuroradiology, neurointerventional radiology

d. interventional radiology

e. musculoskeletal radiology

f. breast, thyroid radiology

g. pediatric radiology

h. health check-up

4. Please select the position you hold in the Radiology department.

a. Professor

b. Associate professor

c. Assistant professor

5. When did you start working in this institution?

a. In 2020

b. In 2021

[Experience with AI]

6. Do you have any educational experience with artificial intelligence?

a. Yes

b. No

7. Do you have any research experience with artificial intelligence?

a. Yes

b. No

8. How much experience do you feel you had with artificial intelligence-based medical devices before working at our hospital? (Please select the appropriate percentage compared to your current experience level by moving the round button.)


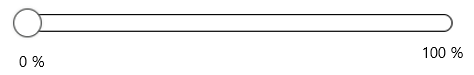


9. How much experience did you gain with artificial intelligence-based medical devices after working at our hospital? (Please select the appropriate percentage by moving the round button.)


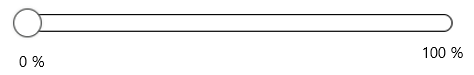


[Utility of the AI-based software]

10. What proportion of all the imaging tests that you are responsible for during a single workday? (Please select the appropriate percentage by moving the round button.)


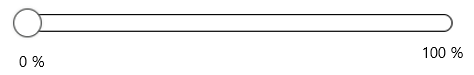


11. Of the chest radiographs you review each day, for what percentage do you check with the AI-based software? (Please select the appropriate percentage by moving the round button.)


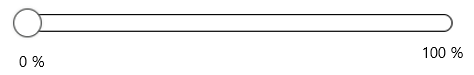


12. Why do you refer to the analysis results of the AI program?

a. Because the AI results are relatively accurate.

b. Because we can lower the risk of missing lesions.

c. Because the user-friendly interface makes it easy to check the AI results.

d. Because it has become routine to check the AI results.

13. If you checked the analysis results of the program, please indicate the results you mainly refer to. (Multiple choice)

a. Overall abnormality score displayed per X-ray

b. Type of lesion detected (abbreviation)

c. Detected lesion location (ROI)

d. Abnormality score of each detected lesion

14. How much trust do you have for the analysis results of the AI program? (Please choose the appropriate percentage by moving the round button.)


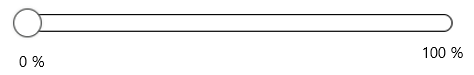


15. Please indicate to what extent your own chest X-ray readings have changed after referring to the AI results. (Please choose the appropriate percentage by moving the round button.)


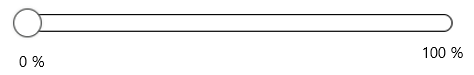


16. How are the chest X-ray reading times affected by this AI program? (-50%: decreased, +50%: increased)


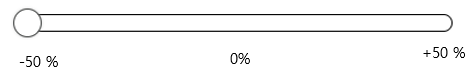


17. Please select what you consider the best advantage of the AI program.

a. Shortens decision times.

b. Helps detect lesions.

c. Helps discriminate normal and abnormal lesions.

d. Helps make a differential diagnosis of lesions.

e. Enables triage of radiographs for reading.

18. How has the number of reading requests for chest radiographs changed after the AI program was applied in our hospital? (-50%: decreased, +50%: increased)


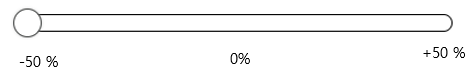


19. Please mark how much the AI program has helped the chest radiograph readings of patients in the following locations.


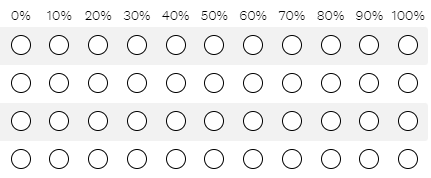


Outpatient

Inpatient

ER

ICU

20. For which finding do you think the AI program is the most useful or expected to be useful? (Please drag and order options according to your perception of its usefulness.)


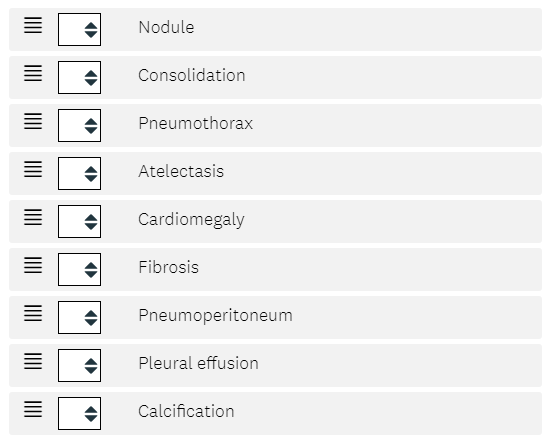


21. Which version of the AI program do you prefer, version 2 or 3?

a. Version 2

b. Version 3

22. Which image do you prefer, the color heatmap or grayscale heatmap?

a. Color heatmap

b. Grayscale heatmap

23. What other information or function would you like to have available in the AI results in the future? (Multiple choice)

a. Addition of readable lesion types

b. Increased accuracy for lesion detection

c. Comparison function that analyzes degrees of change compared to previous images

d. Alarm system for urgent conditions

e. Expansion of the applicable age group

f. Broader application to imagings other than chest imaging

24. After using the AI analysis program, how have your perceptions on AI-based medical devices changed? (-50%: negative, +50%: positive attitude)


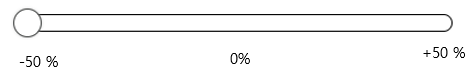


25. Do you plan to refer to AI diagnostic programs for reading in the future? (-50%: negative, +50%: positive attitude)


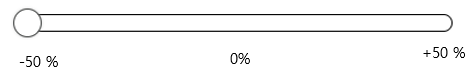


Thank you for participating in our survey.
