# Supplementary material for: Hospital-wide survey of clinical experience with artificial intelligence applied to daily chest radiographs
Source: PLoS One. 2023 Mar 2;18(3):e0282123. doi: 10.1371/journal.pone.0282123 (PMC9980810; doi:10.1371/journal.pone.0282123)
Supplement: S2 File — (DOCX) [file pone.0282123.s002.docx]

**S2 appendix file.** Comparison of responses between staff and trainees among clinicians.

**Table 1.** Demographics of participants

| Survey Questions | | Staff (n=89) | | Trainee (n=19) | | P-value |
| --- | --- | --- | --- | --- | --- | --- |
|  |  |  |  |  |  |  |
|  |  | n | % | n | % |  |
| Sex | Male | 59 | 66.3 | 10 | 52.6 | 0.26^b^ |
|  | Female | 30 | 33.7 | 9 | 47.4 |  |
| Age | 20-29 | 0 | 0 | 10 | 52.6 | <0.001^c^ |
|  | 30-39 | 46 | 51.7 | 9 | 47.4 |  |
|  | 40-49 | 30 | 33.7 | 0 | 0 |  |
|  | 50-59 | 13 | 14.6 | 0 | 0 |  |
| Title | Assistant professor | 44 | 78.6 | 12 | 21.4 | . |
|  | Associate professor | 29 | 80.6 | 7 | 19.4 |  |
|  | Professor | 67 | 85.9 | 11 | 14.1 |  |
|  | Resident, intern | 66 | 92.96 | 5 | 7.04 |  |
| Location of dedicated patients^a^ | ER | 27 | 31.03 | 2 | 11.11 | 0.145^c^ |
|  | ICU | 60 | 68.97 | 16 | 88.89 |  |
| Experience with AI-based research | Inpatient | 21 | 24.14 | 1 | 5.56 | 0.112^c^ |
|  | Outpatient | 66 | 75.86 | 17 | 94.44 |  |

^a^Multiple choice, ^b^Two-sample t-test, ^c^Fisher’s Exact test

Abbreviations: AI = artificial intelligence, ER = emergency room, ICU = intensive care unit.

**Table 2.** Scale bar questions about the utilization of chest radiographs

| Survey Questions | | Staff  (n=65) | | | | | | Trainee (n=13) | | | | | | P-value^b^ |
| --- | --- | --- | --- | --- | --- | --- | --- | --- | --- | --- | --- | --- | --- | --- |
|  |  |  |  |  |  |  |  |  |  |  |  |  |  |  |
|  |  | n | Mean | SD | Median | Q1 | Q3 | n | Mean | SD | Median | Q1 | Q3 |  |
| Proportion of chest radiographs among all utilized images in a day | | 65 | 43.4 | 29.6 | 49 | 12 | 70 | 13 | 54.3 | 26.7 | 60 | 40 | 70 | 0.31 |
| Proportion of cases referring to AI results among all utilized chest radiographs in a day | | 65 | 47.4 | 41.2 | 32 | 7 | 100 | 13 | 38.7 | 41.5 | 15 | 10 | 80 | 0.457 |
| Overall experience with AI | ~2020.2 | 65 | 15.1 | 19.6 | 2 | 0 | 25 | 13 | 6.2 | 12.6 | 0 | 0 | 10 | 0.038 |
|  | 2020.3~ | 65 | 39.4 | 26.1 | 46 | 20 | 52 | 13 | 31.3 | 21.4 | 30.5 | 20 | 50 | 0.212 |
|  | Difference | 65 | 24.3 | 23.2 | 21 | 5 | 43 | 13 | 25.2 | 22.7 | 22 | 10 | 50 | 0.963 |
|  | P-value^a^ |  | <0.001 | |  |  |  |  | <0.001 | |  |  |  |  |

^a^Paired t-test (within Group), ^b^Wilcoxon rank-sum test (between groups).

Data were presented as means with standard deviations (SDs) or medians with interquartile ranges (Q1-Q3).

Abbreviations: AI = artificial intelligence.

**Table 3.** Multiple choice questions about the AI experience

| Survey Questions | | Staff (n=65) | Trainees (n=13) | Overall, % |
| --- | --- | --- | --- | --- |
|  |  | n | n |  |
| Reason for referring to the analysis results of the AI program | Relatively accurate AI results | 12 | 1 | 16.7 |
|  | Lowers the risk of missing lesions | 48 | 7 | 70.5 |
|  | User-friendly method makes checking easy | 20 | 6 | 33.3 |
|  | Has become routine to check the AI results | 15 | 4 | 24.4 |
| AI results that were mainly referred to | Total abnormality score | 31 | 5 | 46.2 |
|  | Lesion type (abbreviation) | 31 | 7 | 48.7 |
|  | Lesion location (ROI) | 43 | 9 | 66.7 |
|  | Abnormality score (per lesion) | 18 | 2 | 25.6 |
| Best advantage of the AI-based software | Shortens decision times | 30 | 5 | 44.9 |
|  | Lesion detection | 41 | 8 | 62.8 |
|  | Discrimination of normal and abnormal lesions | 40 | 5 | 57.7 |
|  | Differential diagnosis of lesions | 10 | 2 | 15.4 |
|  | Triage of radiographs for reading | 3 | 1 | 5.1 |
| AI results that would be welcomed if made available through future developments in technology | Addition of readable lesion types | 13 | 2 | 10.5 |
|  | Increased diagnostic accuracy for lesion detection | 33 | 7 | 36.8 |
|  | Comparison function for lesions | 38 | 8 | 42.1 |
|  | Alarm system for urgent conditions | 38 | 6 | 31.6 |
|  | Expansion of the applicable age range | 10 | 1 | 5.3 |
|  | Broader application to imagings other than chest imaging | 22 | 3 | 15.8 |

Abbreviations: AI = artificial intelligence, ROI = region-of-interest.

| Survey Questions | Scale (%) | Staff (n=65) | | | | | | Trainees (n=13) | | | | | | P-value^a^ |
| --- | --- | --- | --- | --- | --- | --- | --- | --- | --- | --- | --- | --- | --- | --- |
|  |  |  |  |  |  |  |  |  |  |  |  |  |  |  |
|  |  | n | Mean | SD | Median | Q1 | Q3 | n | Mean | SD | Median | Q1 | Q3 |  |
| Extent of changing one’s own reading results  after referring to AI results | 0~100 | 65 | 21.2 | 24.4 | 12 | 0 | 30 | 13 | 19.1 | 14.6 | 20 | 10 | 30 | 0.715 |
| Subjective trust levels for AI results | 0-100 | 65 | 67.0 | 20.2 | 72 | 50 | 80 | 13 | 53.9 | 21.7 | 51 | 50 | 69 | 0.052 |
| Influence on the reading times of chest radiographs | -50~+50 | 65 | -10.7 | 25.8 | -18 | -25 | 7 | 13 | -10.8 | 21.8 | -10 | -23 | 0 | 0.649 |
| Influence on the number of reading requests | -50~+50 | 65 | -13.3 | 18.1 | 0 | -25 | 0 | 13 | -6.9 | 10.5 | 0 | -15 | 0 | 0.36 |
| Diagnostic accuracy of readings | -50~+50 | 65 | 25.6 | 14.3 | 24 | 19 | 34 | 13 | 14.9 | 14.9 | 15 | 10 | 23 | 0.026 |
| Perceptions on AI-based medical devices | -50~+50 | 65 | 29.3 | 16.4 | 30 | 17 | 50 | 13 | 18.8 | 17.6 | 20 | 0 | 25 | 0.049 |
| Perceptions on the future use of AI | -50~+50 | 65 | 33.8 | 14.0 | 30 | 20 | 50 | 13 | 23.8 | 20.4 | 15 | 11 | 50 | 0.089 |

**Table 4.** Scale bar questions about the AI experience

^a^Wilcoxon rank-sum test (between groups)

Data were presented as means with standard deviations (SDs) or medians with interquartile ranges (Q1-Q3).

Abbreviations: AI = artificial intelligence.
